# Supplementary material for: Phenol-chloroform-based RNA purification for detection of SARS-CoV-2 by RT-qPCR: Comparison with automated systems
Source: PLoS One. 2021 Feb 24;16(2):e0247524. doi: 10.1371/journal.pone.0247524 (PMC7904160; doi:10.1371/journal.pone.0247524)
Supplement: S1 Protocol — (PDF) [file pone.0247524.s001.pdf]

S1 Protocol

The acid guanidinium thiocyanate-phenol-chloroform extraction method
200 µl of the sample specimens were aliquoted into sterile 1.5 ml test tubes containing 800 µl of TRI Reagent® (Catalog No. T9424, Sigma-Aldrich). This step was performed under Class II conditions, while the remainder of the RNA extraction, after inactivation of the virus, was performed in a conventional laboratory. Once mixed and transported to the conventional laboratory, the test tubes with sample material and Tri Reagent® were added 200 µl chloroform and mixed by vortexing (5 sec. at max speed). Samples were then incubated for 2 minutes at room temperature and subsequently centrifuged at 14,000g for 15 minutes (4°C). Only the aqueous phase (500 µl) of the resulting mixture located at the top of the tube was processed further, while the remainder of the Tri Reagent®/chlorofom mixture was discarded. The aqueous phase was pipetted into a new tube containing 2 µl of GlycoBlue™ (Catalog No. AM9515 ThermoFisher). 600 µl of isopropanol was then added to the tubes containing the aqueous phase, after which the samples were mixed by vortexing (3 sec at max speed) and then incubated at room temperature (20-25°C) for 20 minutes. The samples were then centrifuged at 14,000g for 15 minutes (4°C), the supernatant removed while making sure the blue RNA pellet remained at the bottom of the tube. The RNA pellets were then washed in 1 ml of 75% Ethanol, vortexed (3 sec. at max speed) and centrifuged at 8,000g for 5 minutes (4°C). The supernatants were then removed while making sure that the RNA pellets remained at the bottom of the tubes. After removal of the Ethanol, samples were incubated at room temperature (20-25°C) for 10 minutes with the lid open to allow excess ethanol to evaporate. Subsequently the RNA pellets were resuspended in 30 µl of RNAse-free water and heated to 60°C for 5 minutes on a heating block. RNA samples were then vortexed 3 sec. and then centrifuged briefly (10 sec. on table centrifuge) to collect all the liquid at the bottom of the tube. Samples were stored at 4°C before transported on ice to the Department of Clinical Microbiology for further analysis.

SARS-CoV-2 RT-qPCR analysis
The SARS-CoV-2 was detected according to the real-time PCR protocol established by Corman et al. (7). Detection of the internal control Newcastle disease virus NDV was performed using primers and probe sequences kindly provided by dr. Kurt J. Handberg, Department of Clinical Microbiology, Skejby, Denmark. In brief, TaqMan™ Fast Virus 1-Step Master Mix (ThermoFisher) was used for the amplification reaction. A final concentration 1000 nmol of primers and 200 nmol of probes were added to a total reaction volume of 20 ul, containing 6 µl of RNA (purified RNA obtained from either Maxwell® automated RNA extraction or from AGPC extraction). Samples were analyzed on a LightCycler®480 II (Roche) using the following program 50°C for 5min, 95°C for 20sec followed by 45 cycles of 95°C for 15sec and 60°C for 1min.
E_Sarbeco_F1: ACAGGTACGTTAATAGTTAATAGCGT
E_Sarbeco_R2: ATATTGCAGCAGTACGCACACA
E_Sarbeco_P1: FAM_ACACTAGCCATCCTTACTGCGCTTCG_BHQ1
NDV-F: 5'-CAC TGT CGG CAT TAT CGA TGA-3’
NDV-R: 5'-GAG CAT CGC AGC GGA AA-3’
NDV-Probe: 5'-FAM-CCC AAG CGC GAG TTA-MGB-3’

Pipeline setup

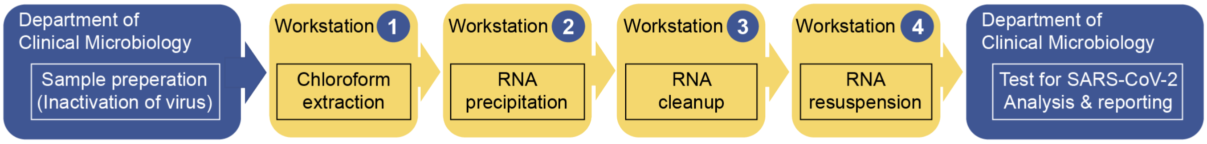

Supplemental Figure 1 Schematic representation of the pipeline setup at the University of Southern Denmark to purify RNA from sample specimens using the AGPC method.

Detailed protocol

Materials

Tri Reagent®	Catalog No. T9424	Merck
GlycoBlue^{TM}	Catalog No. AM9515	Thermofisher
Chloroform	Catalog No. C2432	Merck
2-propanol	Catalog No. 1136	ChemSolute
Ethanol	Catalog No. 1.00983	Merck
RNAse-free water	Catalog No. 129114	Qiagen

- Prepare stocks of chloroform, 2-propanol and 75% Ethanol in suitable 50 ml containers for easy access.
- Prepare RNAse-free water stocks is aliquoted into 1.5 ml sterile test tubes
- Prepare 800µl TRI Reagent aliquoted into 1.5 ml sterile test tubes (rack A)
- Prepare 2 µl GlycoBlue^{TM} aliquoted into1.5 ml sterile test tubes (rack B)

Sample material

At the Department of Clinical Microbiology, 200µl of sample media (obtained from a 1 ml Eswab sample) is aliquoted into the Eppendorf tube with TRI Reagent in rack A and mixed by vortexing. This part is performed solely by personnel at the Department of Clinical Microbiology, which also handles patient/sample registration and coding.

Procedure

1. Test tubes with sample material and TRI Reagent (rack A) are briefly spun down at the start of the procedure (5000g pulse) to collect the material at the bottom of the tubes.
2. 200 µl chloroform is added to the samples in rack A and mixed by vortexing (5 sec, max).
3. Samples are incubated for 2 minutes at room temperature (20-25°C).
4. Samples are centrifuged at 14,000g for 15 minutes (4°C).
5. Check that the tubes in rack A are in the correct order and place them in parallel with the identical numbered test tubes in rack B containing 2µl GlycoBlue^{TM}.
6. Transfer the top aqueous phase (500 µl) from the tubes in rack A to the identically marked tubes in rack B. Double check/verify that the numbered tubes in rack A and B align. Then the test tubes with the remaining material from rack A can be discarded.
7. Add 600 µl of 2-propanol to the samples in rack B and mix by vortexing (3 sec, max).

8. Incubate samples at room temperature (20-25°C) for 20 minutes (15-25 min range).
9. Centrifuge the samples at 14,000g for 15 minutes (4°C). **Note:** Place the hinge of the test tube so it points outwards to simplify localization of RNA pellet after centrifugation.
10. Remove 1ml supernatant (pipette from opposite side of tube hinge). **IMPORTANT:** Identify the blue RNA pellet and make sure the RNA pellet stays at the bottom of the tube.
11. Add 1 ml of Ethanol (75%) and wash the RNA pellet by vortexing (3 sec, max).
12. Centrifuge the samples at 8,000g for 5 minutes (4°C).
13. Carefully remove 1 ml supernatant (pipette from opposite side of tube hinge). **IMPORTANT:** Identify the blue RNA pellet and make sure the RNA pellet stays at the bottom of the tube.
14. Centrifuge the samples briefly at 8,000g for 30 sec at 4°C.
15. Carefully remove ethanol residue with a 200µl pipette. **IMPORTANT:** Identify the blue RNA pellet and make sure the RNA pellet stays at the bottom of the tube.
16. Incubate the samples at room temperature (20-25°C) for 10 minutes with the lid open to allow evaporation of the remaining ethanol.
17. Resuspend the RNA pellet in 30 µl RNAse-free water.
18. Close the lid of the tubes and place the samples on the heat block (60°C) for 5 minutes.
19. Vortex samples for 3 sec. and then centrifuge briefly to collect liquid at the bottom.
20. Place the samples in the fridge (4°C)/ice until collected by the Department of Clinical Microbiology for further analysis.

Notes:

a) Samples are purified in batches of 24 samples (number can be adjusted).
b) The procedure is optimized so that all pipetting steps can be performed in one single motion.
c) The individual steps of handling open samples are carried out in separate fume hoods (4 workstations) if possible, divided as follows:
    1. Adding chloroform and centrifugation with TRI Reagent (Steps 1-4 of the procedure)
    2. Transfer of phase / addition of isopropanol (Steps 5-8 of the procedure)
    3. Wash with 75% ethanol (Steps 10-13 of the procedure)
    4. Drying of RNA / resuspension in RNAse-free water (Steps 15-17 of the procedure)
d) Dividing the procedure into 4 separate workstations reduces the risk of errors and ensures a greater uniformity in sample handling. Workflow chart is attached.
e) The procedure is carried out in series; thus, personnel perform the same steps repetitively at the separate workstations. See procedure outlined below.

Workstation 1 - Chloroform extraction

1. Take test tubes containing sample material and TRI Reagent from the refrigerator. Take 22-24 tubes at a time, starting with tube #1.
2. Test tubes with sample material and TRI Reagent (rack A) are briefly spun down at the start of the procedure (5000g pulse) to collect the material at the bottom of the tubes.
3. 200 µl chloroform is added to test tubes containing sample material and TRI Reagent (rack A) using an electronic pipette. **Important:** Chloroform is volatile and drips, so work quickly, one tube at a time.
4. Mix all samples with chloroform by vortexing. (Vortex samples 2 by 2, 5 sec at max setting).
5. Samples are incubated for 2 minutes at room temperature (20-25°C).
6. Samples are centrifuged at 14,000g for 15 minutes (4°C).
7. While centrifuging the samples, prepare the next batch of 22-24 samples.

Notes:

a) Start a new series of test tubes every 20 minutes to keep the pipeline running steadily.
b) Important: Only work with one open tube at a time.
c) Important: Change the tip between each pipetting.

Workstation 2 – Material transfer & RNA precipitation

1. Check that the tubes in rack A are in the correct order and place them in parallel with the identical numbered tubes in rack B containing GlycoBlue™.
2. Transfer the top aqueous phase (500 µl) from the tubes in rack A to the identically marked tubes in rack B. Double check/verify that the numbered tubes in rack A and B match. Then the test tubes with the remaining material from rack A can be discarded.
3. Add 600 µl of isopropanol to the samples in rack B and mix by vortexing (3 sec vortex - max).
4. Incubate samples at room temperature (20-25°C) for 20 minutes (15-25 min range).
5. Centrifuge the samples at 14,000g for 15 minutes (4°C). **Note:** Place the hinges of the test tubes so they point outward to simplify localization of RNA pellet after centrifugation.

Notes:

a) Transfer aqueous phase for all test tubes first, then reverify the correct order of tubes.
b) Only manual pipette P1000 is used for aspirating aqueous phase.
c) Important: Only work with one open tube (set) at a time when transferring the aqueous phase.
d) Important: Change the tip between each pipetting.

Workstation 3 – Ethanol wash

1. Aspirate 1ml supernatant (pipette from opposite side of tube hinge). **IMPORTANT:** Identify the blue RNA pellet and make sure the RNA pellet stays at the bottom of the tube.
2. Add 1 ml of Ethanol (75%) and place tubes in a new rack.
3. Wash the RNA pellet in the tubes by vortexing (3 sec, max).
4. Centrifuge the samples at 8,000g for 5 minutes (4°C).

Notes:

a) Only use the manual pipette P1000 for aspirating supernatant. Leave any additional residue of liquid and aspirate with the pipette in one motion.
b) Important: Only work with one open tube at a time.
c) Important: Change the tip between each pipetting

Workstation 4 – Ethanol removal and resuspension

1. Carefully remove 1 ml supernatant (pipette from opposite side of tube hinge). **IMPORTANT:** Identify the blue RNA pellet and make sure the RNA pellet stays at the bottom of the tube.
2. Centrifuge the samples briefly at 8,000g for 30 sec at 4°C.
3. Carefully remove ethanol residue with a 200µl pipette. **IMPORTANT:** Identify the blue RNA pellet and make sure the RNA pellet stays at the bottom of the tube.
4. Incubate the samples at room temperature (20-25°C) for 10 minutes with the lid open to allow evaporation of the remaining ethanol.
5. Add 30 µl RNAse-free water to the bottom of test tubes and close the lids.
6. Place the samples on the heat block (60°C) for 5 minutes.
7. Vortex samples 3 sec. and then centrifuge briefly (10 sec) to collect liquid at the bottom.
8. Place the samples in the fridge (4°C)/ice to be collected by the Department of Clinical Microbiology for further analysis.

Notes:

a) Only manual pipettes P1000 / P200 are used for aspirating supernatant.
b) Avoid touching the pellet when adding the 30µl RNAse-free water.
c) Important: Change the tip between each pipetting
