# Supplementary material for: Phenol-chloroform-based RNA purification for detection of SARS-CoV-2 by RT-qPCR: Comparison with automated systems
Source: PLoS One. 2021 Feb 24;16(2):e0247524. doi: 10.1371/journal.pone.0247524 (PMC7904160; doi:10.1371/journal.pone.0247524)
Supplement: Attachment — Submitted filename: Response to reviewers.pdf [file pone.0247524.s002.pdf]

Resubmission of *PLOS One* manuscript PONE-D-20-39657
Dimke et al.
Phenol-chloroform-based RNA purification for detection of SARS-CoV-2 by RT-qPCR: comparison with automated systems

---

Response to Editor and Reviewer's comments

Academic Editor:
1. Please ensure that your manuscript meets PLOS ONE's style requirements, including those for file naming.

- We have formatted and rearranged the manuscript to meet all PLOS ONE's requirements.

2. To meet PLOS ONE submission guidelines, in your Methods section, please provide additional information regarding your statistical analyses.

- We have added additional information regarding statistical analyses used to the Methods section. **These changes can be found on line 119-129.**

3. Your ethics statement should only appear in the Methods section of your manuscript. If your ethics statement is written in any section besides the Methods, please move it to the Methods section and delete it from any other section. Please ensure that your ethics statement is included in your manuscript, as the ethics statement entered into the online submission form will not be published alongside your manuscript.

- We have moved the Ethics statement to the Methods section of the manuscript. **These changes can be found on line 131-134.**

Reviewer #1:
This is a focused and straightforward examination of the efficacy of the guanidinium thiocyanate-phenol-chloroform (AGPC) method of RNA extraction for RNA sample preparation used in the detection of SARS-CoV-2 by RT-qPCR. The primary objective of the authors was to determine whether the much less expensive and technologically simpler, although more labor intensive, bench top method of RNA isolation from nasopharyngeal and oropharyngeal swabs is as effective as automated RNA isolations carried out by two different instruments currently in use for COVID-19 testing. The rational for this study is that evidence supporting efficacy of the AGPC extraction method can enable COVID-19 testing to be carried out in locations that lack the resources for expensive, high-throughput RNA isolation devices. The results clearly show that the bench top method can reliably be used in place of automated instruments.
The study is technically sound and well controlled, and the data is appropriately analyzed for statistical significance. The experiments and results are thoroughly and clearly described in well-written, grammatically correct English.

- We thank the Reviewer for their kind review.

Reviewer #2:
The manuscript entitled "Phenol-chloroform-based RNA purification for detection of SARS-CcV-2 by RT qPCR: comparison with automated systems" is a valuable contribution to the field of SARS-CoV-2 detection systems.

However, although conventional RNA purification can be a valid alternative to commercial platforms for RNA extraction, it is also true that in a pandemic setting when every moment matters, the use of commercial kits and automated system for the detection of SARS-COv-2 can speed up the identification process of SARS-CoV-2

positive subjects. Maybe the Authors should discuss this point better in their manuscript.

- We appreciate and agree with the Reviewer's suggestion. We have included a short discussion of the benefits of automated systems for rapid identification of SARS-CoV-2 positive subjects during a pandemic. **These additions can be found on line 205-207 and line 222-255.**

Line 135: in the Materials and Methods section, the Authors indicated that both oropharyngeal and nasopharyngeal swabs were collected. Please make the required correction.

- We have corrected this error. **The addition can be found on line 198-199**

Reviewer #3:

In this paper by Moeller lab, they compare the result of the standard phenol choloform isolation of SARS-CoV-2 RNA to automated RNA extraction systems. They use qRT-PCR to detect the inactivated viral RNA and compare the two methods head to head. Appropriate positive controls are used. They find that the PC method is comparable to the automated systems in terms of true positive (TP) TN FP FN. Importantly a detailed protocol is presented in the supplemental data. While this method is typically successfully performed by those skilled in working with RNA and therefore more human error could be introduced during the isolation, it does offer an alternative to automated systems in developing countries that may not have access to those equipment. While PC extraction of SARS-CoV-2 RNA has been preciously reported (their ref 5) this study for the first times does a head to head comparison of the two methods and shows them to be equivalent.

- We thank the Reviewer for their kind comments.
